# Transoral Endoscopic and Minimally Invasive Thyroidectomy

**DOI:** 10.1001/jamasurg.2025.3248

**Published:** 2025-09-03

**Authors:** Ting-Chun Kuo, Kuen-Yuan Chen, Chieh-Wen Lai, Ming-Tsan Lin, Chin-Hao Chang, Ming-Hsun Wu

**Affiliations:** 1 Department of Surgery, National Taiwan University Hospital, Taipei, Taiwan; 2 School of Medicine, Tzu Chi University, Hualien, Taiwan; 3 Department of Surgery, Taipei Tzu Chi Hospital, Buddhist Tzu Chi Medical Foundation, New Taipei, Taiwan; 4 Department of Medical Research, National Taiwan University Hospital & National Taiwan University, Taipei, Taiwan

## Abstract

**Importance:**

The transoral endoscopic thyroidectomy vestibular approach (TOETVA) is a scarless surgical technique gaining popularity; however, its safety, cost, and impact on pathological evaluation compared with minimally invasive nonendoscopic thyroidectomy (MINET) remain under evaluation.

**Objective:**

To compare surgical outcomes, pathological considerations, and cost profiles following TOETVA vs MINET using a propensity-matched cohort.

**Design, Setting, and Participants:**

This cohort study included 720 patients undergoing oncoplastic thyroidectomy between January 2021 and January 2023, with 12-month follow-up, at a tertiary referral center in Taipei, Taiwan. Propensity score matching was used to generate balanced cohorts of 210 patients each.

**Main Outcomes and Measures:**

Primary outcomes included operative duration (measured from incision to closure), intraoperative neuromonitoring (IONM) alerts (recorded in real time via standard IONM systems), parathyroid preservation (assessed by identification and in situ preservation), postoperative pain (evaluated using the visual analog scale [VAS] score on postoperative day 1), specimen integrity (as documented in final pathology reports), postoperative complications, and overall cost (derived from itemized hospital billing records).

**Results:**

Among 420 matched patients (mean age [SD], 45.6 [12.3] years; 371 [88.3%] female), TOETVA was associated with longer mean (SD) operative time (127.9 [43.8] vs 68.1 [23.3] minutes; mean difference, 59.73 minutes; 95% CI, 53.20-66.26; *P* < .001), fewer IONM alerts (4/255 [1.6%] vs 13/262 [5.0%]; risk difference, −3.39%; 95% CI, −6.54 to 0.08; *P* = .04), and fewer inadvertently resected parathyroid glands (24/510 [4.7%] vs 57/524 [10.9%]; risk difference, −6.17%; 95% CI, −9.42 to −2.85; *P* < .001). Pain scores were slightly higher in TOETVA (mean [SD] VAS score: 0.88 [0.22] vs 0.38 [0.13]; mean difference, 0.50; 95% CI, 0.47-0.54; *P* < .001). Specimen disruption was more frequent with TOETVA (27/210 [12.9%] vs 8/210 [3.8%]; risk difference, 9.05%; 95% CI, 3.52 to 14.28; *P* = .008). Total costs were higher in TOETVA (mean [SD], $4680 [328] vs $2734 [296]; mean difference, $1946; 95% CI, 1885.19 to 2006.87; *P* < .001).

**Conclusions and Relevance:**

In this propensity-matched cohort study, TOETVA was associated with favorable cosmetic outcomes and improved nerve and parathyroid preservation but longer operative times, higher costs, and greater risk of specimen disruption. These findings support the need for careful patient selection and further prospective evaluation.

## Key Points

**Question:**

How does the transoral endoscopic thyroidectomy vestibular approach (TOETVA) compare with minimally invasive nonendoscopic thyroidectomy (MINET) in terms of surgical outcomes, safety, cost, and pathological integrity?

**Findings:**

In a propensity score–matched cohort of 420 patients, TOETVA was associated with significantly longer operative and preparation times and higher overall costs. However, TOETVA demonstrated fewer intraoperative neuromonitoring alerts and a lower incidence of inadvertent parathyroid resection; TOETVA was also associated with a higher rate of specimen disruption.

**Meaning:**

Although TOETVA is a cosmetically favorable and safe alternative to MINET, with potential benefits in nerve and parathyroid preservation, it may increase operative demands and pose challenges in pathological evaluation, underscoring the importance of careful patient selection.

## Teaser

This cohort study evaluates surgical outcomes, pathological considerations, and cost profiles of the transoral endoscopic thyroidectomy vestibular approach compared to minimally invasive nonendoscopic thyroidectomy.

## Introduction

Various surgical techniques exist to minimize or conceal neck scars during thyroidectomies.^1,2^ Among these, minimally invasive nonendoscopic thyroidectomy (MINET) has emerged as the most widely adopted true minimally invasive procedure.^3,4^ MINET refines a conventional thyroidectomy by using a smaller anterior cervical incision, thereby preserving familiar anatomical orientation while reducing tissue trauma and improving cosmetic outcomes. A relatively recent innovation is the transoral endoscopic thyroidectomy vestibular approach (TOETVA),^5,6,7,8^ which meets the demand for scarless outcomes and provides shorter access to the thyroid compared to other endoscopic thyroidectomy approaches.

A previous large-scale study^9^ demonstrated that TOETVA resulted in lower pain scores on the visual analog scale (VAS) compared to conventional thyroidectomy, suggesting its viability as a minimally invasive procedure. However, while most surgeons tend to minimize transcervical incisions in line with MINET principles, this approach inevitably results in a smaller dissection area compared to TOETVA. For a fair comparison, both procedures should use identical instruments (including intraoperative neuromonitoring [IONM] and energy devices), and the criteria should account for equivalent tumor sizes.

TOETVA involves specimen removal through a narrow tract, which increases the risk of tumor breakage and may complicate pathological evaluation.^10^ Furthermore, many endocrine surgeons interested in adopting TOETVA lack access to information about its potential systemwide impact on hospital operations. This study aims to compare cohorts undergoing MINET or TOETVA to address these concerns.

## Methods

### Patient Eligibility and Study Design

This cohort study followed the Strengthening the Reporting of Observational Studies in Epidemiology (STROBE) reporting guideline. Medical records were retrospectively collected from patients undergoing MINET and TOETVA procedures at National Taiwan University Hospital, a tertiary referral medical center, between January 2021 and January 2023, with 12-month postoperative follow-up. This study was approved by the National Taiwan University Hospital research ethics committee and registered with ClinicalTrials.gov (NCT04569513). Because of the retrospective nature of the study, the requirement for informed consent was waived by the institutional review board.

The sex of study participants was recorded as male or female based on medical records. Gender identity was not assessed, as it was not relevant to the study objectives. Race and ethnicity data were not collected or reported in this study, as they were not relevant to the study objectives and not routinely documented in the medical records.

### Anesthesia

For TOETVA, nasotracheal intubation with IONM electrodes was used to optimize operative field exposure and address disinfection concerns while providing face protection. For MINET, orotracheal intubation with IONM electrodes was used.

Pain was managed with remifentanil via target-controlled infusion adjusted to vital signs. In both groups, cisatracurium besylate, a nondepolarizing muscle relaxant, was titrated using neuromuscular block monitoring to maintain an ideal train-of-4 ratio of 40% (TOF-Watch).^11^ This facilitated nerve identification and enabled smooth IONM throughout the procedures.

### Surgical Technique and Neuromonitoring

For TOETVA, we implemented the procedure and postoperative management protocol as described by Anuwong.^5,9^ For MINET, we followed the procedure outlined by Linos,^3^ performing surgery through a cervical incision less than 3.5 cm. Magnifying loupes were not routinely used in MINET, as sufficient visualization was consistently achieved under direct vision. All patients in both groups were scheduled for discharge on postoperative day 1.

Neuromonitoring was conducted using either the NIM-Response 3.0 System (Medtronic) or the C2 System (Inomed Medizintechnik). All procedures adhered to the International Neural Monitoring Study Group guidelines for IONM.^12^

No drain was placed in either group. All patients received a standardized postoperative nonopioid pain control regimen consisting of oral acetaminophen, 500 mg, 4 times daily for 4 days and a single dose of diclofenac sodium, 100 mg, administered on the operative day.

### Neuromonitoring Signal Interpretation

We routinely documented intraoperative loss of signal and mechanisms of recurrent laryngeal nerve injury. Following initial identification of the recurrent laryngeal nerve, a 1.0-mA current was applied for precise localization, and the resulting electromyographic signal was recorded as the R1 signal. Intermittent IONM was used, with alert conditions defined as a 50% or greater reduction in electromyographic amplitude relative to the baseline R1 signal.^13^ In the event of an alert, the corresponding intraoperative event was carefully analyzed to determine the neuromonitoring pattern associated with the electromyographic change.

### Definition of Resected Parathyroid

All resected thyroid specimens were examined using a near-infrared autofluorescence imaging device, the SURGIMAGE SIM 1000H (SurgImage Corporation), for systematic data acquisition.^14^ Areas showing sharp contrast against surrounding tissue were identified as potential parathyroid glands. These autofluorescence-positive regions were marked, resected, and confirmed through tissue washout parathyroid hormone analysis. Confirmed parathyroid tissues that were inadvertently resected were considered adverse events and were subsequently reimplanted.

### Outcome Evaluation

Time in the operating room refers to the total duration of the patient’s presence in the operating theater. Preparation time includes anesthetic induction, patient positioning, disinfection, and draping. Operative time is defined as the interval from surgical incision to wound closure.

Postoperative pain was assessed using a VAS ranging from 0 to 10.^15^ VAS pain scores were recorded at 3 fixed postoperative time points: on arrival to the recovery room, and at 8 and 16 hours postoperatively. For analysis, the mean of the 3 VAS scores was calculated for each patient to represent their overall postoperative pain experience.

### Safety

Vocal cord function was routinely evaluated using ultrasonography, with direct laryngoscope examination performed when symptoms were suspected. Vocal cord palsy or hypoparathyroidism was considered permanent if persisting beyond 12 months.

### Costs

Cost analysis was conducted at 3 months postoperatively. All costs were converted from Taiwanese dollars (TWD) to US dollars at a rate of 1 TWD to $0.0333. Overall costs encompassed medical expenses for operations and hospitalizations. Operation costs included procedure fees, medical equipment, and medications as determined by Taiwan’s National Health Insurance Administration Ministry of Health and Welfare. Hospitalization costs covered inpatient care, including any readmissions for complications.

### Statistical Analysis

The data are summarized using descriptive statistics. Categorical variables are shown as frequencies and percentages, while continuous variables are displayed as means with SDs. Comparisons were analyzed using independent *t* test for continuous variables and χ^2^ test or Fisher exact test for categorical variables, as appropriate.

Propensity scores were estimated using logistic regression incorporating baseline covariates. To reduce confounding bias due to baseline differences between groups, a 1:1 nearest-neighbor greedy matching algorithm without replacement was applied, using a caliper width of 0.1 standard deviations of the logit of the propensity score. Patients outside the region of common support were excluded from the matched analysis. Covariate balance was assessed using standardized mean differences (SMDs), with a threshold of ≤0.1 indicating adequate balance.

All variables were complete with no missing data. After matching, continuous outcomes were analyzed using paired *t* tests. For binary categorical outcomes, the McNemar test was used to account for paired data. For categorical comparisons, such as histopathological diagnoses, risk differences and 95% CIs were calculated using Wald or Newcombe hybrid score methods, depending on event frequency. Fisher exact test was used for comparisons with expected counts less than 5. Subgroup analyses were also conducted among patients with follicular neoplasms to assess differences in capsule integrity. All statistical tests were 2-sided, and a *P* value <.05 was considered statistically significant. Statistical analyses were performed using SAS software version 9.4 (SAS Institute).

## Results

### Patient Demographic and Clinical Characteristics

**Figure 1.**
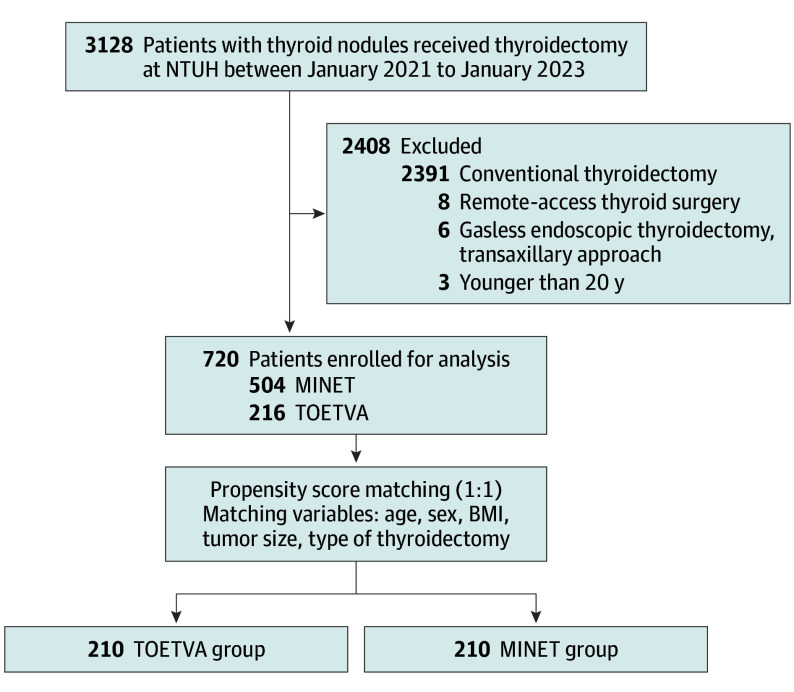
Flow Diagram of Patient Selection and Propensity Score Matching MINET indicates minimally invasive nonendoscopic thyroidectomy; NTUH, National Taiwan University Hospital; TOETVA, transoral endoscopic thyroidectomy vestibular approach. See the eFigure in Supplement 1 for the full distribution plots.

Among 3114 thyroidectomies performed during the study period, 720 patients (mean [SD] age, 45.6 [12.3] years; 371 [88.3%] female and 120 [16.7%] male) underwent oncoplastic thyroidectomies for cosmetic purposes. From this cohort, patients were categorized into 2 groups based on the surgical approach: 216 in the TOETVA group and 504 in the MINET group (Figure 1).

Before matching, patients in the TOETVA group were significantly younger, included fewer men, had lower body mass index (BMI), and had smaller dominant tumors than those in the MINET group. Additionally, a higher proportion of patients in the TOETVA group underwent unilateral thyroidectomy compared to those in the MINET group (Table 1). These demographic differences reflect the inherent selection bias associated with oncoplastic thyroidectomy preferences.

**Table 1.** Demographic and Clinical Characteristics of Patients Before and After Propensity Score Matching

Characteristic	Before matching				After matching			
	TOETVA (n = 216)	MINET (n = 504)	*P* value^a^	SMD^b^	TOETVA (n = 210)	MINET (n = 210)	*P* value^a^	SMD^b^
Age, mean (SD), y^c^	44.6 (11.8)	50.5 (13.1)	<.001	0.47	45.1 (11.7)	46.2 (12.9)	.36	0.09
Sex, No. (%)^d^								
Female	190 (88.0)	410 (81.4)	.03	0.18	184 (87.6)	187 (89.1)	.65	0.04
Male	26 (12.0)	94 (18.7)			26 (12.4)	23 (11.0)		
BMI, mean (SD)^c^	22.9 (3.6)	24.1 (4.0)	<.001	0.32	23.0 (3.6)	23.2 (3.5)	.54	0.04
Tumor size, mean (SD) cm^d^^,^^e^	2.5 (1.8)	3.0 (2.1)	<.001	0.29	2.5 (1.8)	2.5 (1.6)	.85	0.02
Malignancy, No. (%)^b^	82 (38.0)	194 (38.5)	.90	0.01	79 (37.6)	82 (39.0)	.76	0.03
Unilateral thyroidectomy, No. (%)^d^	169 (78.2)	355 (70.4)	.03	0.18	165 (78.6)	158 (75.2)	.42	0.03
Prior neck procedure or radiation, No. (%)^c^	0	2 (0.4)	>.99	0.09^f^	0	1 (0.5)	>.99^f^	0.10
Cardiovascular disease, No. (%)^c^	0	3 (0.6)	.56	0.11^f^	0	0	NA	NA
Diabetes, No. (%)^c^	1 (0.5)	3 (0.6)	>.99	0.02^f^	1 (0.5)	0	>.99^f^	0.10

Abbreviations: BMI, body mass index (calculated as weight in kilograms divided by height in meters squared); MINET, minimally invasive nonendoscopic thyroidectomy; NA, not applicable; SMD, standardized mean difference; TOETVA, transoral endoscopic thyroidectomy vestibular approach.

^a^ A 2-sided *P* value <.05 was considered statistically significant.

^b^ SMDs were calculated to assess covariate balance between groups, with values ≤0.1 indicating adequate balance after propensity score matching.

^c^ Compared using independent *t* tests.

^d^ Compared using χ^2^ or Fisher exact tests as appropriate.

^e^ Tumor size refers to maximal tumor diameter.

^f^ Fisher exact test used.

After propensity score matching for age, sex, BMI, tumor size, and type of thyroidectomy, the baseline characteristics between the 2 groups were balanced, as demonstrated in Table 1 and the eFigure in Supplement 1. Figure 1 outlines the patient selection process and highlights the application of propensity score matching to create comparable cohorts of 210 patients each, ensuring balanced baseline characteristics for meaningful comparisons.

### Surgical Outcomes

TOETVA has significantly longer operative duration (mean [SD], 127.9 [43.8] vs 68.1 [23.3] minutes; mean difference, 59.73 minutes; 95% CI, 53.20-66.26; *P* < .001), preparation, and total operating room time than MINET (Table 2). The overall complication rate was comparable between the 2 groups (TOETVA: 8/210 [3.8%] vs MINET: 13/210 [6.2%]; *P* = .26), including rates of vocal cord palsy (3/210 [1.4%] vs 5/210 [2.4%]; *P* = .72) and hypoparathyroidism (4/210 [1.9%] vs 7/210 [3.3%]; *P* = .55). All vocal cord palsy cases were confirmed by fiberoptic laryngoscopy postoperatively. There were no cases of mortality or conversion in the TOETVA group or wound extension in the MINET group (Table 2).

**Table 2.** Intraoperative Parameters and Surgical Outcomes in Transoral Endoscopic Thyroidectomy Vestibular Approach (TOETVA) vs Minimally Invasive Nonendoscopic Thyroidectomy (MINET)

Characteristic	TOETVA (n = 210)	MINET (n = 210)	Mean or risk difference (95% CI)^a^	*P* value^b^
Time in operating room, mean (SD), min^c^	182.8 (44.3)	94.2 (23.0)	88.50 (81.93 to 95.11)	<.001
Preparation time	36.5 (10.3)	22.0 (1.9)	14.52 (13.12 to 15.91)	<.001
Operative time	127.9 (43.8)	68.1 (23.3)	59.73 (53.20 to 66.26)	<.001
Overall complications, No. (%)^d^	8 (3.8)	13 (6.2)	−2.38 (−6.73 to 2.13)	.26
Vocal cord palsy	3 (1.4)	5 (2.4)	−0.95 (−3.96 to 2.26)	.72
Transient	2 (1.0)	4 (1.9)	−0.95 (−3.67 to 2.02)	.68
Permanent	1 (0.5)	1 (0.5)	0.00 (−2.21 to 2.21)	>.99
Hypoparathyroidism	4 (1.9)	7 (3.3)	−1.43 (−4.79 to 2.15)	.55
Transient	3 (1.4)	5 (2.4)	−0.95 (−3.96 to 2.26)	.72
Permanent	1 (0.5)	2 (1.0)	−0.48 (−2.75 to 2.01)	>.99
Hematoma requiring reoperation	1 (0.5)	1 (0.5)	0.00 (−2.21 to 2.21)	>.99
Conversion or wound extension	0	0	0.00 (−1.80 to 1.80)	NA
IONM alert (signal reduction >50%), No./nerve at risk (%)^d^	4/255 (1.6)	13/262 (5.0)	−3.39 (−6.54 to 0.08)	.04^e^
Traction related	1/255 (0.4)	9/262 (3.4)	−3.04 (−5.46 to −0.06)	.02^e^
Thermal related	2/255 (0.8)	3/262 (1.2)	−0.36 (−2.53 to 1.88)	>.99^e^
Transection	1/255 (0.4)	1/262 (0.4)	0.01 (−1.81 to 1.79)	>.99^e^
Inadvertently resected parathyroid, No./glands encountered (%)^d^	24/510 (4.7)	57/524 (10.9)	−6.17 (−9.42 to −2.85)	<.001
VAS pain score, mean (SD)^c^	0.9 (0.2)	0.4 (0.1)	0.50 (0.47 to 0.54)	<.001
Hospital stay, mean (SD), d^c^	2.0 (0.2)	2.0 (0.1)	0.02 (−0.0042 to 0.05)	.10
Overall cost, mean (SD), TWD^c^	140 546 (9847)	82 107 (8883)	58 439.00 (56 612.42 to 60 266.37)	<.001
Overall cost, mean (SD), $^c^	4680 (328)	2734 (296)	1946.00 (1885.19 to 2006.87)	<.001
Hypertrophic scar at 12 mo, No. (%)^d^	0	6 (2.9)	−2.86 (−5.20 to 0.38)	NA

Abbreviations: IONM, intraoperative nerve monitoring; NA, not applicable; TWD, new Taiwan dollar; VAS, visual analog scale.

^a^ Mean differences and 95% CIs were calculated using paired *t* tests. Risk differences and 95% CIs were calculated for categorical outcomes.

^b^ A 2-sided *P* value <.05 was considered statistically significant.

^c^ Compared using paired *t* tests.

^d^ Compared using the McNemar test for paired binary outcomes. For outcomes measured at the organ level (eg, nerves at risk or glands encountered), χ^2^ test or Fisher exact test was used as appropriate.

^e^ Fisher exact test used.

TOETVA demonstrated a fewer alert incidence of intraoperative nerve (signal reduction >50%) (4/255 [1.6%] vs 13/262 [5.0%]; risk difference, −3.39%; 95% CI, −6.54 to 0.08; *P* = .04) compared to MINET. TOETVA also demonstrated a lower traction-related IONM alert rate (1/255 [0.4%] vs 9/262 [3.4%]; *P* = .02) (Table 2). The inadvertently resected parathyroid rate was higher in the MINET group than in the TOETVA group (57/224 [10.9%] vs 24/510 [4.7]%; risk difference, −6.17%; 95% CI, −9.42 to −2.85; *P* < .001).

**Figure 2.**
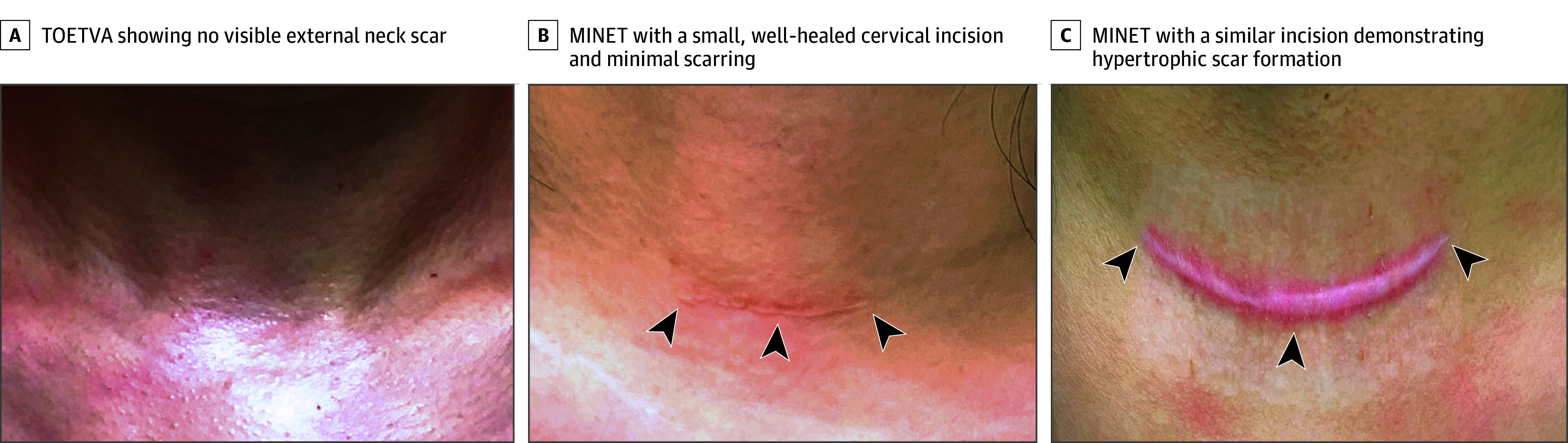
Postoperative Neck Appearance Following the Transoral Endoscopic Thyroidectomy Vestibular Approach (TOETVA) and Minimally Invasive Nonendoscopic Thyroidectomy (MINET) at 12 Months, With and Without Hypertrophic Scarring

Postoperative mean (SD) pain scores were higher in TOETVA patients (VAS: 0.88 [0.22] vs 0.38 [0.13]; mean difference, 0.50; 95% CI, 0.47-0.54; *P* < .001) (Table 2). No patient required postoperative additional intravenous analgesia beyond our pain protocol. Regarding wound appearance in MINET, the incidence of hypertrophic scarring at 12 months postoperatively was 6 of 210 patients (2.86%) (Figure 2).

TOETVA was associated with higher overall costs (mean [SD], $4680 [328] vs $2734 [296]; mean difference, $1946; 95% CI, 1885.19 to 2006.87; *P* < .001).

### Histopathological Findings

The distribution of histopathological diagnoses for both groups is presented in Table 3. Adenomatous goiter was the most common benign condition. The incidence of well-differentiated tumor of uncertain malignant potential was higher in the TOETVA group (10/210 [4.8%] vs 3/210 [1.4%]) compared to MINET group (*P* = .049).

**Table 3.** Histopathological Findings and Specimen Integrity in Transoral Endoscopic Thyroidectomy Vestibular Approach (TOETVA) and Minimally Invasive Nonendoscopic Thyroidectomy (MINET)

Variable	TOETVA (n = 210)	MINET (n = 210)	Risk difference (95% CI)^a^^,^^b^	*P* value^c^
**Histopathological finding, No. (%)**				
Adenomatous goiter	86 (41.0)	92 (43.8)	−2.86 (−12.26 to 6.47)	.55
PTC	64 (30.5)	78 (37.1)	−6.67 (−15.71 to 2.22)	.69
Follicular/Hürthle cell adenoma	32 (15.2)	31 (14.8)	0.48 (−6.41 to 7.36)	.89
NIFTP	3 (1.4)	2 (1.0)	0.48 (−2.3 to 3.1)	>.99^d^
FTC	4 (1.9)	3 (1.4)	0.48 (−2.56 to 3.4)	>.99^d^
WDT-UMP	10 (4.8)	3 (1.4)	3.33 (−0.56 to 6.78)	.049
**Specimen integrity, No. (%)**				
Specimen disrupted	27 (12.9)	8 (3.8)	9.05 (3.52 to 14.28)	.001
Incomplete tumor capsule integrity	7 (3.3)	1 (0.5)	2.86 (−0.55 to 5.62)	0.07^d^
FN with incomplete tumor capsule integrity (No./total FN, %)^e^	4/49 (8.2)	1/39 (2.6)	5.60 (−5.63 to 17.31)	0.38^d^

Abbreviations: FN, follicular neoplasm; FTC, follicular thyroid carcinoma; NIFTP, noninvasive follicular thyroid neoplasm with papillarylike nuclear features; PTC, papillary thyroid cancer; WDT-UMP, well-differentiated tumor of uncertain malignant potential.

^a^ Risk differences and 95% CIs were calculated using Wald or Newcombe hybrid score methods, depending on event frequency. Fisher exact test was used for comparisons with expected counts less than 5.

^b^ Comparisons between matched groups were performed using χ^2^ or Fisher exact tests as appropriate.

^c^ A 2-sided *P* value <.05 was considered statistically significant.

^d^ Fisher exact test used.

^e^ FN includes follicular/Hürthle cell adenoma, NIFTP, FTC, and WDT-UMP.

Specimen disruption, based on final pathological assessment, was more common in the TOETVA group compared to the MINET group (27/210 [12.9%] vs 8/210 [3.8%]; risk difference, 9.05%; 95% CI, 3.52 to 14.28; *P* = .001). Tumor capsule integrity was incomplete in 7 TOETVA cases (3.3%) and 1 MINET case (0.5%) (*P* = .07). Among follicular neoplasms—where capsule integrity may influence diagnostic accuracy—incomplete capsule integrity was observed in 4 of 49 TOETVA cases (8.2%) and 1 of 39 MINET cases (2.6%), with no significant difference (*P* = .38).

## Discussion

This cohort study compared TOETVA and MINET procedures, focusing on safety, cost, and pathological considerations. Propensity score matching was used to minimize selection bias. All patients underwent routine neuromonitoring, and postoperative specimens were examined using near-infrared autofluorescence, enabling a direct comparison between techniques.

Thyroidectomy remains the primary therapeutic approach for symptomatic or malignant thyroid nodules, with resultant neck scarring being a major patient concern.^16,17,18,19^ Minimally invasive techniques such as MINET^3^ and minimally invasive video-assisted thyroidectomy,^20^ which use cervical incisions no longer than 3.5 cm, offer comparable cosmetic outcomes and recovery processes.^4^ The newer TOETVA procedure^5,6,7,8,9^ best fulfills the requirement for scarless outcomes, providing shorter access to the thyroid compared to other endoscopic thyroidectomy approaches.^21^ However, questions remain about whether TOETVA can match MINET’s safety profile and minimal invasiveness, while considerations about its overall impact on hospital systems warrant further evaluation.

The results of this study showed no significant differences in overall complication rates between the 2 procedures. Anatomical and physiological preservation of the recurrent laryngeal nerve is crucial in thyroid surgery. Previous studies of TOETVA that lacked the use of routine IONM reported transient and permanent recurrent laryngeal nerve injury rates of approximately 4.3% and 0.1%, respectively.^22^ In our series, both TOETVA and MINET demonstrated low hoarseness rates compared to previous reports.^21,23,24^ The routine use of IONM is increasingly recognized as a valuable real-time adjunct, enhancing the accuracy of recurrent laryngeal nerve identification and functional integrity monitoring during thyroidectomy.^6,25^

All patients in both groups underwent surgery using a standard endotracheal electromyographic tube under direct laryngoscopic visualization. Tube position was carefully verified before anesthesia induction and reconfirmed after patient positioning to ensure consistent and optimal electrode contact with the vocal cords. Baseline electromyographic signals (R1) were routinely obtained and monitored throughout the procedure. No systematic discrepancies in signal detection were observed that could be attributed to tube placement or malpositioning. IONM alerts were most commonly observed during dissection and exploration, particularly during medial traction.^26^ These alerts reflect real-time neural stress or potential compromise. Notably, traction-related IONM alerts occurred significantly more frequently in the MINET group compared to the TOETVA group. This difference may be attributable to the limited visualization and restricted working space inherent to the MINET technique, as opposed to the enhanced magnification and angled dissection trajectory offered by the 30° endoscope used in TOETVA. Endoscopic advantages facilitate precise recurrent laryngeal nerve dissection with less medial traction. The apparent discrepancy between intraoperative IONM alerts and postoperative vocal cord outcomes in the current study suggests the nuanced relationship between neural signal changes and functional recovery. Many IONM alerts likely reflect transient mechanical stress or minor irritation, from which the nerve can fully recover. IONM is intentionally designed with high sensitivity to detect early, potentially reversible changes in nerve function, serving as an early warning system rather than a definitive predictor of permanent injury.^13^

In our study, transient hypoparathyroidism rates were comparable between the TOETVA and MINET groups. However, the MINET group showed a higher incidence of inadvertently resected parathyroid tissue. The enhanced visualization offered by endoscopic techniques enabled better identification and preservation of smaller parathyroid glands. Furthermore, the lateral and posterior endoscopic views improved visualization of posterior structures, facilitating their preservation.^4,9^ Despite this significant difference in inadvertent parathyroid excision rates, no corresponding difference in hypoparathyroidism rates was observed, likely due to the compensatory function of the remaining intact parathyroid glands. Thus, while inadvertent parathyroid excision reflects a measurable technical difference between the 2 approaches, its clinical impact on overall parathyroid function preservation appears limited. Routine postoperative specimen examination using near-infrared autofluorescence, combined with tissue parathyroid hormone washout confirmation, provided objective evaluation of resected parathyroid gland incidence. This protocol also enabled prompt reimplantation of inadvertently excised parathyroid tissue when detected, potentially reducing the risk of permanent hypoparathyroidism.

In this study, the TOETVA group demonstrated a significantly longer operative time compared to the MINET group, consistent with previous studies comparing TOETVA to conventional thyroidectomy.^9,27^ The operative time for MINET in our series was notably shorter than previously reported,^3,4^ likely due to the routine use of energy devices. The need for flap dissection and the creation of a working space inherently prolong the operative time. The TOETVA group also exhibited a longer preparation time, primarily due to additional steps, such as the transition from orotracheal to nasotracheal intubation with a nerve integrity monitoring tube, meticulous facial area protection, and the setup of extra equipment. These preparatory measures differ significantly from those required in cervical approaches. Although orotracheal tube use in TOETVA has been reported,^28,29^ disinfection around the orotracheal tube area remains challenging; therefore, nasotracheal intubation remains our standard practice for TOETVA. The prolonged preparation time underscores increased demands on both personnel and institutional resources beyond the surgical team. Additionally, the extended time in operation room may delay operating room turnover during peak hours, potentially reducing surgical throughput and contributing to intangible institutional costs. As expected, the TOETVA group incurred higher overall costs, consistent with findings from previous studies.^30^

We found that postoperative VAS pain scores were significantly higher in the TOETVA group compared to the MINET group. Interestingly, previous studies have reported lower mean VAS pain scores for TOETVA compared to conventional thyroidectomy.^9^ The lower pain scores observed in our MINET group are likely attributable to the smaller skin incision and the limited extent of flap dissection. Notably, our comparison provides more robust evidence than prior studies, as both groups received identical postoperative pain management protocols, ensuring consistency in analgesic administration and pain control measures.

Our findings demonstrated a higher rate of specimen disruption and tumor capsule damage in the TOETVA group compared to the MINET group, consistent with prior reports.^10,31^ Since these parameters are essential for determining appropriate treatment and follow-up strategies, this limitation warrants careful consideration during patient selection for TOETVA.^32^ Additionally, fragmentation may create a false impression of microscopic extrathyroidal extension or raise suspicion of its presence, potentially altering clinical management.^33^ According to the American Joint Committee on Cancer 8th edition guidelines,^16^ fragmented or disrupted specimens may hinder accurate evaluation of macroscopic extrathyroidal extension. Furthermore, because subcentimeter papillary thyroid cancers often go undetected preoperatively and are found incidentally, every TOETVA procedure carries an inherent risk that specimen fragmentation may reveal an incidental malignancy that cannot be pathologically assessed with confidence.^10,34^ This could lead to diagnostic uncertainty and complicate decisions regarding adjuvant treatment or surveillance.

Specimens disruption often occurs during endoscopic dissection or transoral extraction.^6,35^ Accurate differentiation between benign and malignant follicular neoplasms depends on the identification of capsular or vascular invasion. However, the traction applied during specimen retrieval in TOETVA may compromise capsular integrity, potentially obscuring or destroying key diagnostic features. Although the incidence of well-differentiated tumors of uncertain malignant potential was higher in the TOETVA group compared to the MINET group, our results did not demonstrate a significantly increased incidence of tumor rupture in follicular neoplasms—an event that may complicate diagnosis. Nonetheless, this issue warrants ongoing attention, and the potential for clinically meaningful differences should not be overlooked simply due to the absence of statistical significance. These findings may reflect low event rates combined with limited sample size and statistical power, rather than a true lack of difference between surgical approaches.

### Limitations

Our study has several limitations. First, due to its retrospective design, we were unable to comprehensively evaluate overall resource utilization. Costs related to equipment maintenance, sterilization, and indirect factors were not captured, as our analysis primarily focused on direct procedural and hospitalization expenses. Varying health care systems potentially may limit generalizability.

We also did not account for potential second operations or their associated clinical and economic challenges. Moreover, variations in pathological characteristics may also arise from the specific technique and individual performing the procedure. Our study population had a relatively lower BMI compared to some populations, which may limit the generalizability of our findings to patients with higher BMI, where surgical complexity and incision requirements could differ substantially. Detailed analyses comparing patient characteristics across different surgical approaches are still pending, highlighting the need for further evidence. To more thoroughly assess the long-term safety and cost-effectiveness of these procedures, multicenter prospective studies with extended follow-up are warranted.

## Conclusions

TOETVA provides a cosmetically superior, safe alternative to MINET, with reduced risk of parathyroid gland injury and fewer IONM alerts. However, it is associated with longer operative times, increased resource utilization, and higher rates of specimen disruption that may complicate pathological assessment. While not all outcomes reached statistical significance, observed differences in parameters may still carry meaningful clinical implications. particularly in cases such as capsular disruption in follicular neoplasms. These observations underscore the need for careful patient selection and larger prospective studies to validate the long-term safety, pathological adequacy, and cost implications of TOETVA.

## Notes

Supplement 1. eFigure. Balance of Covariates Before and After Matching

Supplement 2. Data sharing statement
