# Supplementary material for: Transoral Endoscopic and Minimally Invasive Thyroidectomy
Source: JAMA Surg. 2025 Sep 3;160(11):1203–10. doi: 10.1001/jamasurg.2025.3248 (PMC12409647; doi:10.1001/jamasurg.2025.3248)
Supplement: Supplement 1. — eFigure. Balance of Covariates Before and After Matching [file jamasurg-e253248-s001.pdf]

Supplemental Online Content

Kuo TC, Kuen-Yuan Chen KY, Lai CW, Lin MT, Chang CH, Wu MH. Transoral endoscopic and minimally invasive thyroidectomy. *JAMA Surg*. Published online September 3, 2025. doi:10.1001/jamasurg.2025.3248

eFigure. Balance of Covariates Before and After Matching

This supplemental material has been provided by the authors to give readers additional information about their work.

© 2025 Kuo TC et al. *JAMA Surgery*.

eFigure. Balance of Covariates Before and After Matching

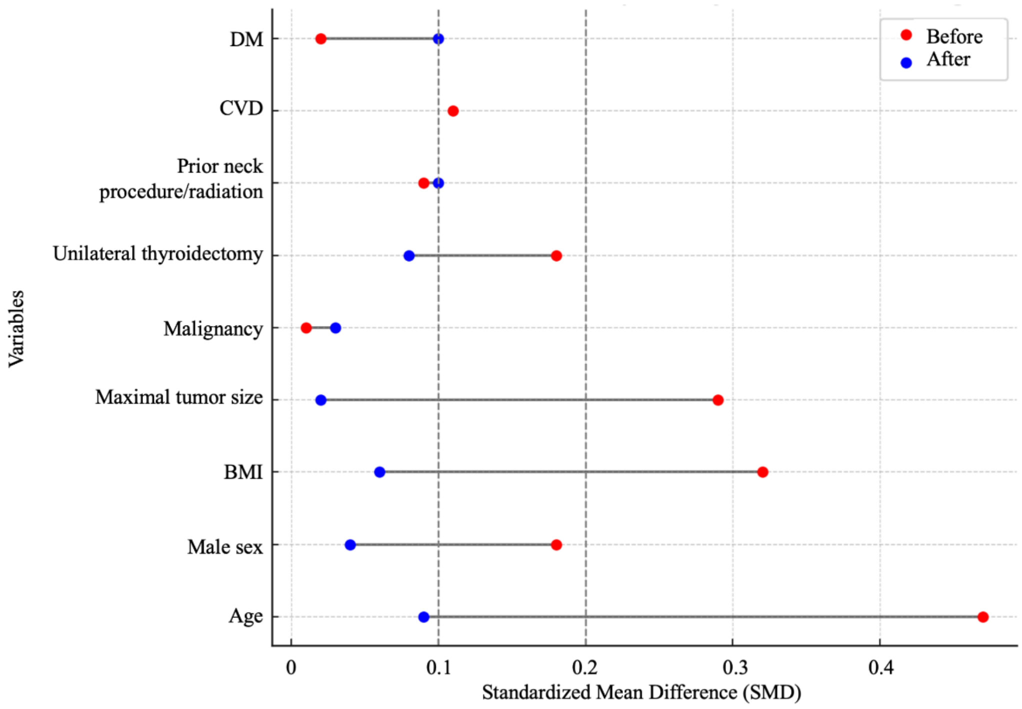

© 2025 Kuo TC et al. *JAMA Surgery*.
