# Supplementary material for: Transoral Endoscopic and Minimally Invasive Thyroidectomy
Source: JAMA Surg. 2025 Sep 3;160(11):1203–10. doi: 10.1001/jamasurg.2025.3248 (PMC12409647; doi:10.1001/jamasurg.2025.3248)
Supplement: Supplement 2. — Data sharing statement [file jamasurg-e253248-s002.pdf]

Data Sharing Statement

Kuo. Transoral Endoscopic and Minimally Invasive Thyroidectomy. *JAMA Surg*. Published September 03, 2025. doi:10.1001/jamasurg.2025.3248

Data
Data available: Yes
Data types: Data (not involving human participants)
How to access data: Interested researchers may contact the corresponding author, Dr. Ming-Hsun Wu, at dtsurgp9@gmail.com.
When available: With publication

Supporting Documents
Document types: Statistical/analytic code
How to access documents: Deidentified individual participant data (IPD), study protocol, and statistical analysis code will be made available. Interested researchers may contact the corresponding author, Dr. Ming-Hsun Wu, at dtsurgp9@gmail.com.
When available: With publication

Additional Information
Who can access the data: Requests for access to the data will require submission of a methodologically sound research proposal and approval by the corresponding author. Data Additional Information: Clinical Trials.gov (NCT04569513).
Types of analyses: The data will be made available for academic, non-commercial research purposes only, particularly for meta-analyses, surgical outcomes comparisons, or methodology validation studies.
Mechanisms of data availability: Data will be provided with investigator support, after approval of a methodologically sound proposal, and with a signed data access agreement.
